# Looking Beyond Family for Support and Intimacy: How Older Single and Widowed Black Women Cope With Loneliness

**DOI:** 10.1093/geroni/igab046.1545

**Published:** 2021-12-17

**Authors:** Margaret Salisu

**Affiliations:** SUNY@Downstate, Brooklyn, New York, United States

## Abstract

Older adults are at risk of depression and higher rates of suicide due to loneliness. Loneliness is even more pronounced for single and bereaved older adults. Although loneliness is one of the benchmarks for measuring well-being, little is known about how older Black women understand and cope with loneliness. The aim of this qualitative phenomenological study is to explore how older Black women understand and cope with loneliness. Fourteen older single and/or widowed Black women in New York City participated in this study. The application of the Black feminist standpoint theory helped to understand the loneliness of the participants in the context of their Blackness. Three themes emerged from the study: loneliness increasing with age, looking beyond the family for intimacy, and balance. All the participants expressed feeling some degree of loneliness, regardless of whether they lived alone or with family. Although they had robust social circles, they experienced loneliness, feelings of isolation, and a loss of emotional connection and intimacy. However, these losses went unexpressed, as the participants struggled to balance their position as Black matriarchs—which they considered an important familial role due to their identity as older Black women—with their emotional needs. These two roles did not converge for the women, as the role of Black matriarch posed an expectation they would not experience emotional loss in old age. The implications of this study for policy and practice pertain to the intersection of race, age, gender, and sexuality in assessing the risk of loneliness.

